# Supplementary material for: Longitudinal Outcomes of Gender Identity in Children (LOGIC): study protocol for a retrospective analysis of the characteristics and outcomes of children referred to specialist gender services in the UK and the Netherlands
Source: BMJ Open. 2021 Nov 10;11(11):e054895. doi: 10.1136/bmjopen-2021-054895 (PMC8587379; doi:10.1136/bmjopen-2021-054895)
Supplement: Supplementary data [file bmjopen-2021-054895supp001.pdf]

Appendix 1. Information regarding appointment types in the specialist gender clinics

	Type of clinical appointment	Definition
T&P NHSFT		
	1: Early Liaison with endocrine clinic for <15 yrs[a]	Appointment with the endocrinology clinic for those aged 15+ years
	2: Liaison with endocrine clinic for 15+ yrs[a]	Appointment with the endocrinology clinic for those aged <15 years
	3: GIDS Outreach Assessment[b]	Assessment undertaken remotely from the main clinic base in London
	4: GIDS Standard Assessment[b]	Assessment which took place at the London clinic
	5: GIDS Treatment Outreach[c]	Psychosocial treatment undertaken remotely from the main clinic base in London
	6: GIDS Treatment Standard[c]	Psychosocial treatment which took place at the London clinic
	7: GIDS Young Persons Group[d]	Group appointment specifically for young people (take place during GIDS group sessions)
	8: Group (not specified)[d]	Group appointment (take place during GIDS group sessions)
	9: GIDS Transitions Appointment[c]	Appointment for young people with GIDS and GIC clinician to discuss transition to adult service
	10: Endocrinology (15+ yrs)[a]	Appointment with the endocrinology clinic for those aged 15+ years
	11: Endocrinology (<15 yrs)[a]	Appointment with the endocrinology clinic for those aged <15 years
	12: Child, Young Adults and Families (CYAF) Assessment[b]	Assessment by CYAF clinician
	13: CYAF Individual therapy once per week[c]	Individual therapy by a CYAF clinician
	14: CYAF Family Therapy[c]	Family therapy by a CYAF clinician
AUMC		
	1: Intake Psychology[b]	First GID appointment with the psychologist during the diagnostic phase, 60 min
	2: Consultation Psychology[b]	Standard GID assessment with the psychologist during the diagnostic phase, 60 min

3: Psychological Assessment[b]	Appointment for completing questionnaires & psychiatric assessment / interview during the diagnostic phase, 2-3h
4: Screening Psychiatry[b]	First appointment after referral to the AUMC is with the main practitioner / treatment provider<br>For children/adolescents the appointed main practitioner in the hospital is the Child and Adolescent psychiatrist<br>After the psychiatric screening, the psychiatrist refers the CYP to a psychologist for the GID assessment / diagnostic phase
5: Consultation Psychiatry[b]	Psychiatric assessment during the diagnostic phase<br>Usually upon request from psychologist (for example in case when CYP presents with (complex) psychiatric comorbidity)
6: New patient endocrinology[a]	First appointment with the endocrinology clinic for CYP
7: Consultation endocrinology[a]	Standard appointment with the endocrinology clinic for CYP
8: Group consultation endocrinology / Webinar[a]	Group consultation before start medical treatment (providing education/information re medical treatment)
9: DEXA scan[a]	Order for DEXA scan to check bone health
10: Labs[a]	Appointment with the nurse for bloods / weight / height measurements and standard health checks
11: Consultation fertility[a]	Appointment with the fertility doctor (GYN)

Note. Appointment type groupings: [a]Endocrinology/Medical; [b]Assessment; [c]Psychosocial Treatment; [d]Group
