# Supplementary material for: Longitudinal Outcomes of Gender Identity in Children (LOGIC): study protocol for a retrospective analysis of the characteristics and outcomes of children referred to specialist gender services in the UK and the Netherlands
Source: BMJ Open. 2021 Nov 10;11(11):e054895. doi: 10.1136/bmjopen-2021-054895 (PMC8587379; doi:10.1136/bmjopen-2021-054895)
Supplement: Supplementary data [file bmjopen-2021-054895supp002.pdf]

Appendix 2. Information regarding care pathways

In the UK, following the NHS England service specification, all patients attend the service for assessment in the first instance. According to the service specifications, this typically consists of 3-6 appointments, with the number of appointments based on individual need, agreed with the young person and their family. At the end of the assessment, if the young person remains in contact with the service they either continue to explore their gender identity and options around this, or they may be referred to the endocrine clinic with ongoing exploration of gender and pathways and psychosocial support. The current NHS service specification can be found here: https://www.england.nhs.uk/wp-content/uploads/2017/04/gender-development-service-children-adolescents.pdf. Please note that this is currently undergoing a scheduled review, and an amendment was made to the service specification in December 2020 following the Bell vs. Tavistock judicial review. The amendment can be found here: https://www.england.nhs.uk/wp-content/uploads/2020/12/Amendment-to-Gender-Identity-Development-Service-Specification-for-Children-and-Adolescents.pdf

As GIDS is a national service, ‘outreach’ assessment simply refers to the assessment being undertaken remotely from the main clinic base in London. After an assessment, if the CYP remains in contact with the service, appointment type then changes to ‘treatment’: ‘treatment outreach’ would indicate the treatment took place outside the main London clinic base, whereas ‘treatment standard’ would mean that the treatment took place at the London clinic. We will certainly provide additional descriptions when reporting findings.

In the Netherlands, the first consultation (‘intake’) is always with a child and adolescent psychiatrist. The following appointments during the assessment phase are with a psychologist. This usually consists of 3-6 appointments (on a monthly basis), with the number of appointments based on individual need, and agreed with the young person and

their family. At the end of the assessment, the majority of CYP are referred to endocrinology and also continue to see a psychologist for psychosocial support. The young person and their family are seen by the endocrinologist and psychologist every 3 months.
