# Supplementary material for: Longitudinal Outcomes of Gender Identity in Children (LOGIC): study protocol for a retrospective analysis of the characteristics and outcomes of children referred to specialist gender services in the UK and the Netherlands
Source: BMJ Open. 2021 Nov 10;11(11):e054895. doi: 10.1136/bmjopen-2021-054895 (PMC8587379; doi:10.1136/bmjopen-2021-054895)
Supplement: Author's manuscript [file bmjopen-2021-054895.draft_revisions.pdf]

BMJ Open is committed to open peer review. As part of this commitment we make the peer review history of every article we publish publicly available.

When an article is published we post the peer reviewers' comments and the authors' responses online. We also post the versions of the paper that were used during peer review. These are the versions that the peer review comments apply to.

The versions of the paper that follow are the versions that were submitted during the peer review process. They are not the versions of record or the final published versions. They should not be cited or distributed as the published version of this manuscript.

BMJ Open is an open access journal and the full, final, typeset and author-corrected version of record of the manuscript is available on our site with no access controls, subscription charges or pay-per-view fees (http://bmjopen.bmj.com).

If you have any questions on BMJ Open's open peer review process please email info.bmjopen@bmj.com

BMJ Open

Longitudinal Outcomes of Gender Identity in Children (LOGIC): study protocol for a retrospective analysis of the characteristics and outcomes of children referred to specialist gender services in the UK and the Netherlands

Journal:	*BMJ Open*
Manuscript ID	bmjopen-2021-054895
Article Type:	Protocol
Date Submitted by the Author:	28-Jun-2021
Complete List of Authors:	Kennedy, Eilis; Tavistock and Portman NHS Foundation Trust, Research & Development; University College London, Research Department of Clinical, Educational and Health Psychology<br>Lane, Chloe; Tavistock and Portman NHS Foundation Trust, Research & Development<br>Stynes, Hannah; Tavistock and Portman NHS Foundation Trust<br>Ranieri, Veronica; Tavistock and Portman NHS Foundation Trust, Research & Development; University College London, Science & Technology Studies<br>Spinner, Lauren; Tavistock and Portman NHS Foundation Trust, Research & Development<br>Carmicheal, Polly; Tavistock and Portman NHS Foundation Trust, Gender Identity Development Service<br>Omar, Rumana; University College London,<br>Vickerstaff, Victoria; University College London, UCL Mental Health Sciences Unit<br>Hunter, Rachael; University College London, Research Dept of Primary Care and Population Health<br>Senior, Robert; Tavistock and Portman NHS Foundation Trust<br>Butler, Gary; University College London Hospital, Paediatrics and Adolescents<br>Baron-Cohen, Simon; University of Cambridge<br>De Graaf, Nastasja; Amsterdam University Medical Centres<br>Steensma, Thomas; Amsterdam University Medical Centres, Center of Expertise on Gender Dysphoria<br>de Vries, Annelou; VU Medisch Centrum, Child and Adolescent Psychiatry<br>Young, Bridget; University of Liverpool<br>King, Michael; University College Medical School, Division of Psychiatry
Keywords:	Child & adolescent psychiatry < PSYCHIATRY, Sexual and gender disorders < PSYCHIATRY, Health economics < HEALTH SERVICES ADMINISTRATION & MANAGEMENT

SCHOLARONE™
Manuscripts

I, the Submitting Author has the right to grant and does grant on behalf of all authors of the Work (as defined in the below author licence), an exclusive licence and/or a non-exclusive licence for contributions from authors who are: i) UK Crown employees; ii) where BMJ has agreed a CC-BY licence shall apply, and/or iii) in accordance with the terms applicable for US Federal Government officers or employees acting as part of their official duties; on a worldwide, perpetual, irrevocable, royalty-free basis to BMJ Publishing Group Ltd ("BMJ") its licensees and where the relevant Journal is co-owned by BMJ to the co-owners of the Journal, to publish the Work in this journal and any other BMJ products and to exploit all rights, as set out in our licence.

The Submitting Author accepts and understands that any supply made under these terms is made by BMJ to the Submitting Author unless you are acting as an employee on behalf of your employer or a postgraduate student of an affiliated institution which is paying any applicable article publishing charge ("APC") for Open Access articles. Where the Submitting Author wishes to make the Work available on an Open Access basis (and intends to pay the relevant APC), the terms of reuse of such Open Access shall be governed by a Creative Commons licence – details of these licences and which Creative Commons licence will apply to this Work are set out in our licence referred to above.

Other than as permitted in any relevant BMJ Author's Self Archiving Policies, I confirm this Work has not been accepted for publication elsewhere, is not being considered for publication elsewhere and does not duplicate material already published. I confirm all authors consent to publication of this Work and authorise the granting of this licence.

For peer review only

Longitudinal Outcomes of Gender Identity in Children (LOGIC): study protocol for a retrospective analysis of the characteristics and outcomes of children referred to specialist gender services in the UK and the Netherlands

Eilis Kennedy[1,2]; Chloe Lane[1]; Hannah Stynes[1]; Veronica Ranieri[1]; Lauren Spinner[1]; Polly Carmichael[3]; Rumana Omar[4]; Victoria Vickerstaff[5,12]; Rachael Hunter[5]; Rob Senior[1]; Gary Butler[3,6]; Simon Baron-Cohen[7]; Nastasja M. de Graaf[8,9]; Thomas D. Steensma[8,9]; Annelou L.C. de Vries[8,10]; Bridget Young[11]; Michael King[12]

[1] Research & Development Unit, Tavistock Centre, Tavistock and Portman NHS Foundation Trust

[2] Research Department of Clinical, Educational and Health Psychology, University College London

[3] Gender Identity Development Service, Tavistock and Portman NHS Foundation Trust

[4] Department of Statistical Science, University College London

[5] Research Department of Primary Care and Population Health, Royal Free Medical School, University College London

[6] Department of Paediatric and Adolescent Endocrinology, University College London Hospital and University College London, Great Ormond Street Institute of Child Health

[7] Autism Research Centre, Department of Psychiatry, University of Cambridge

[8] Center of Expertise on Gender Dysphoria, Amsterdam University Medical Centres

For peer review only

LOGIC retrospective analysis protocol

[9] Department of Medical Psychology, Amsterdam University Medical Center

[10] Department of Child and Adolescent Psychiatry, Amsterdam University Medical Center

[11] Institute of Population Health Sciences, University of Liverpool

[12] Division of Psychiatry, Faculty of Brain Sciences, University College London

Address for correspondence: Dr Eilis Kennedy, Research & Development Unit, Tavistock Centre, Tavistock & Portman NHS Foundation Trust, 120 Belsize Lane, NW3 5AS, United Kingdom (e-mail: ekennedy@tavi-port.nhs.uk).

For peer review only

Abstract

Introduction

Specialist gender services for children and young people (CYP) worldwide have experienced a significant increase in referrals in recent years. As rates of referrals increase, it is important to understand the characteristics and profile of CYP attending these services in order to inform treatment pathways and to ensure optimal outcomes.

Methods and Analysis

A retrospective observational study of clinical health records from specialist gender services for CYP in the UK and the Netherlands. The retrospective analysis will examine routinely collected clinical and outcome measures data including demographic, clinical, gender identity-related, and healthcare resource use information. Data will be reported for each service and also compared between services. This study forms part of a wider programme of research investigating outcomes of gender identity in children (the LOGIC study).

Ethics and Dissemination

The proposed study has been approved by the UK Health Research Authority and London – Hampstead Research Ethics Committee as application 19/LO/0181. The study findings will be published in peer-reviewed journals and presented at both conferences and stakeholder events.

For peer review only

Article Summary

Strengths and limitations

- This study will involve a retrospective analysis of routinely collected data from two European specialist gender services in a large cohort of CYP aged ≤13 years.
- A detailed evaluation of service use and costs will be ascertained for specialist gender services for CYP in both the UK and the Netherlands.
- As data from two distinct specialist gender services (in the UK and the Netherlands) will be utilised, not all variables will be available for CYP from both services.
- As the study will use data extracted from clinical health records, there will inevitably be some missing data but this will be taken into account in the analyses.
- Selection of measures and variables is constrained to those that are routinely collected by the services but limitations of these will be discussed when interpreting the findings.

For peer review only

Introduction

In recent years, specialist gender services for CYP have experienced a significant rise in referrals worldwide (1,2). For example, the gender identity development service (GIDS) at the Tavistock & Portman NHS Foundation Trust (NHSFT) in the UK has reported a 382% increase from 678 referrals in 2014/15 to 2,590 referrals in 2018/19 (3). Time trends in relation to the profile of CYP referred to services have also been noted. In particular, there has been a shift in recent years to an increase in referrals of CYP assigned female at birth (4,5). However, a recent study of time trends in adolescent referrals in the Netherlands found that, other than a shift in sex ratio, no other time trends were observed in relation to demographics or intensity of gender dysphoria (6). As rates of referrals increase, it is important to understand the characteristics and profile of CYP attending these services in order to inform treatment pathways and to ensure optimal outcomes for these CYP.

The specialist gender services at the Tavistock & Portman NHSFT in the UK and Amsterdam University Medical Centre (AUMC) in the Netherlands are two of the longest established and largest services in Europe for CYP seeking support in relation to their gender identity. Both of the services follow a similar assessment protocol and both have consistently used the same outcome measures over the past 8 years, enabling comparisons to be made between the services. These include a multi-disciplinary clinical assessment and completion of the Child Behaviour Checklist (CBCL), the Youth Self Report (YSR) and the Teacher Report Form (TRF) (7) to assess emotional and behavioural functioning, and the Social Responsiveness Scale (SRS) (8) to assess autistic traits. Data from these services have previously been compared and some cross-cultural differences in the nature of referrals to

For peer review only - http://bmjopen.bmj.com/site/about/guidelines.xhtml

these clinics have previously been reported. For example, a recent study comparing psychological functioning in adolescents (aged 12-18 years) referred to specialist gender services across four European countries (including the UK and the Netherlands) identified that, at the time of referral, emotional and behavioural problems and peer relationship difficulties were most prevalent in CYP presenting to services in the UK (9). Conversely, these issues were least prevalent in CYP presenting to services in the Netherlands. In addition, a greater number of younger children presenting to specialist gender services who have already made changes in their clothing, hairstyle, first name and pronouns to reflect their gender identity (sometimes referred to as social transition) at the time of referral has been reported(10). Recent research has suggested that making these changes early on can have desirable outcomes for CYP (11–13). However, research on this matter in pre-pubertal children is limited, and thus we need to know more about how making such changes in dress and behaviour relates to later outcomes (14). This study will extend current understanding of the nature of CYP referrals, particularly in relation to younger children, by providing further opportunity to characterise the profile and outcomes of attendees at each service, whilst also exploring potential cross-cultural differences.

Several longitudinal prospective cohort studies of CYP attending specialist gender services are now ongoing (15,16). However, retrospective analysis of clinical data can provide important and unique insights into the characteristics and outcomes of CYP referred to these services which are not yet available from these ongoing prospective studies. This is particularly pertinent as it is widely acknowledged that the evidence base on which current treatment protocols is based is limited (14). Furthermore, retrospective studies include a whole specified cohort, which is not necessarily feasible within prospective studies which generally require a process of recruitment and novel data collection. Retrospective studies of clinical cohorts therefore provide a valuable and informative addition to the literature.

For peer review only - http://bmjopen.bmj.com/site/about/guidelines.xhtml

As much of the existing literature has focused on adolescents, little is currently known about the overall characteristics and outcomes of younger CYP, particularly pre-pubertal and early pubertal children, who attend specialist gender services. Consequently, there is limited evidence to inform the likely trajectories and outcomes of these CYP and to enable clinical care pathways to be tailored accordingly. The present study aims to address this gap in the literature by profiling CYP aged ≤13 years who first attended specialist gender services across an eight year period (2009-2017). It will describe and, where possible, compare the outcomes of CYP attendees in relation to their demographic and family backgrounds, emotional and behavioural functioning, autistic traits and gender identity (e.g. diagnosis of gender dysphoria and social transition). Measures relating to emotional and behavioural functioning and autistic traits will be included, as mental health conditions and autism have been reported to co-occur for some CYP who are referred to specialist gender services (17–20). Differences in referrals and treatment pathways in each country will be explored e.g. numbers of children presenting to the clinic who have already socially transitioned and age at time of referral to paediatric endocrinology. Service use and outcomes will be identified, particularly in relation to CYP who attend a paediatric endocrinology clinic and those who do not. A healthcare resource costing of both services will also be undertaken. It is anticipated that the proposed research will improve understanding of the characteristics of service users in order to help in the planning and organisation of services and to address the need for tailored support when required.

Aims

This study aims to identify 1) the profile of CYP aged ≤13 years attending specialist gender services in the UK and the Netherlands between 2009 and 2017; 2) the proportion of these CYP who a) experience gender dysphoria, b) socially transition, c) access medical treatment (e.g. hormone blockers and cross sex hormones) and d) have co-occurring autistic

For peer review only - http://bmjopen.bmj.com/site/about/guidelines.xhtml

traits; 3) the profile of CYP who attend paediatric endocrinology clinics and the profile of CYP who do not attend these clinics; 4) the service use and costs of CYP attending specialist gender services; 5) costs for CYP who attend paediatric endocrinology clinics and costs for CYP who do not attend these clinics.

Methods and Analysis

Study design

A retrospective observational study of clinical health records from specialist gender services in the UK and the Netherlands across an eight year period (2009-2017). See Table 1 for a full list of variables and measures. This study forms part of a wider programme of research investigating outcomes of gender identity in children (the LOGIC study) (21). This programme of research utilises a mixed methods approach, incorporating both quantitative and qualitative longitudinal studies to investigate the experiences, outcomes and wellbeing of families referred to the UK GIDS.

[Insert Table 1 about here]

Study population

The study population will consist of all CYP aged ≤13 years who attended at least one appointment at a specialist gender service (GIDS or AUMC) between 2009 and 2017 and were recorded in the electronic patient records system used by the services. This will include approximately 1040 CYP from GIDS and 529 CYP from AUMC. CYP with differences in sex development and those referred to the service to obtain support with a parent undergoing gender transition will be excluded from the analyses.

For peer review only - http://bmjopen.bmj.com/site/about/guidelines.xhtml

LOGIC retrospective analysis protocol

Data source

The study will consist of a retrospective analysis of routinely collected clinical data extracted from both the Tavistock & Portman NHSFT and the AUMC in the Netherlands between 2009 and 2017. The UK GIDS was established in 1989 and is currently one of the largest, if not the largest, specialist gender clinic for CYP in the world. It is a nationally commissioned service covering England, Wales, Northern Ireland and in part, Scotland and the Republic of Ireland, through a series of outreach clinics and a main hub in London. The Center of Expertise on Gender Dysphoria in the Netherlands was established in 1988, and is one of the oldest and most established clinics. These two sites were chosen as they represent two of the largest and longest serving specialist clinics in Europe for CYP seeking support relating to their gender identity. These services are therefore uniquely placed to undertake a retrospective analysis of the characteristics and outcomes of CYP who attended the clinics across an eight year period. Completion of assessment and outcome measures such as the CBCL, YSR, TRF, and SRS was entirely voluntary and not a condition of receiving care. The data from these measures were collected retrospectively for this study.

Procedure

The research teams at each respective site (the Tavistock & Portman NHSFT in the UK and AUMC) will submit a request for data extraction to their local informatics team. Data will be extracted by each local informatics team via their electronic service user record software and entered into a CSV-formatted dataset. Members of the research teams at each respective site will manually input any data into the datasets which cannot be extracted by the informatics teams, such as handwritten or typed information obtained from assessment reports. Data for social transition will be hand-searched by the UK research team from medical records, although the content of which can vary enormously from patient-to-patient and by clinician. All identifiable data will be held in a password-protected database on an

For peer review only

encrypted NHS server at the Tavistock and Portman NHSFT until the dataset has been finalised and is ready for analysis. Once the dataset is complete, all identifiable data (NHS patient IDs, dates of birth and all free-text responses) will be removed. The anonymised dataset will then be uploaded and stored onto an encrypted and GDPR-compliant data portal (Data Safe Haven) so that the statistical team at University College London (UCL) PRIMENT's Clinical Trials Unit can access the dataset for analysis. A data sharing agreement will be in place between the Tavistock & Portman NHSFT and the AUMC, each as data controllers. The sites will share ownership of the anonymised datasets once analysis is complete and these datasets will be retained for no longer than 20 years. A collaboration agreement will also be enforced between all participating sites, identifying UCL as the data processor. The data processor shall destroy the data upon request by the Tavistock & Portman NHSFT. The study will run for approximately 2 years (2019 – 2021).

Analysis plan

Data will be analysed in STATA by the statistical team at UCL PRIMENT's Clinical Trials Unit. Characteristics of the CYP will be described using mean (SD), median (IQ range) or frequencies (proportion), as appropriate. In order to address study aim (2), the proportion of CYP who experience gender dysphoria, socially transition, access physical/medical treatment (i.e. attend a paediatric endocrinology clinic) and have co-occurring autistic traits (as measured by the SRS) will be estimated, along with their 95% confidence intervals. In order to address study aim (3), descriptive characteristics of the CYP who attend a paediatric endocrinology clinic will be compared to those for CYP who do not attend such a clinic. Regression models will be used to examine factors that are associated with referral to a paediatric endocrinology clinic. Factors which are likely to be explored within these models include: a) family composition, b) social transition, c) emotional and behavioural functioning

For peer review only - http://bmjopen.bmj.com/site/about/guidelines.xhtml

of CYP (measured by CBCL, YSR and TRF) and d) autistic traits (measured by the SRS). Regression analyses will be adjusted for year of referral.

In relation to study aim (4), the average number of contacts with the service will be reported for each type of appointment. To establish healthcare resource costs, data pertaining to individual appointments (i.e. assessment appointment; psychosocial treatment appointment; group appointment; and endocrine appointment) from the time of referral until discharge from the service will be analysed. These will be costed based on information provided by the service on the number, profession and grade of clinical involvement for each appointment type and using information from the Personal Social Service Resource Unit (PSSRU) to calculate cost per minute (22). The cost per minute for each appointment type will then be multiplied by the duration of appointment, as recorded in patient files. We will conduct a sensitivity analysis using only service provided costs and only NHS Reference Costs (23). The Netherlands will be costed based on PSSRU costing, with a sensitivity analysis using Netherlands specific wages. Costed clinic appointments will then be summed together to calculate the total cost of care for each CYP and divided by contact time to adjust for patients with longer follow-ups. Average total costs of care and appointments will be reported for young people who attended a paediatric endocrinology clinic versus those who did not. These will be reported separately for the UK and the Netherlands.

In order to address study aim (5), information such as (i) costs of care; (ii) country of care; (iii) care pathway (the type of treatment and/or support that the CYP receives throughout their time with the service); (iv) outcomes including wellbeing (measured by CBCL, YSR and TRF); and (v) potential predictors of costs and outcomes (e.g. age at first appointment, gender dysphoria diagnosis and autistic traits) will be used to explore differences in costs between CYP who attend a paediatric endocrinology clinic and those who do not. The purpose of this analysis is to calculate the cost of care for each CYP from the

For peer review only

beginning to end of their time with GIDS, and then report if different CYP have different costs because (i) of the country they are in; (ii) they followed the endocrine pathway (or didn't); (iii) other clinical factors such as the prevalence of autistic traits. Further information regarding care pathways is described in Appendix 1.

All of the analyses will be presented individually for each service and also combined where possible. Where appropriate, analyses will be reported by year (2009 to 2017). If necessary, previously identified differences in baseline presentation of CYP referrals to the two services will be taken into account in the analyses, as well as other differences between the clinics such as the time (year) at which early physical interventions are offered. Potential bias due to missing data will be investigated by comparing the characteristics of CYP who have completed the reported outcome measures to those who have incomplete or no outcome data. Outcome measure data (i.e. CBCL, TRF, YSR, and SRS) will only be included in analyses when ≥70% of the cohort have completed the measures.

Patient and public involvement statement

The LOGIC study was developed in collaboration with UK GIDS users. The research proposal was also discussed at a stakeholder event involving trans youth organisations. The LOGIC study has a patient and public involvement (PPI) group, comprised of parents and CYP who are participating in our ongoing longitudinal cohort study. Findings and outputs will be discussed with the study PPI group.

Ethics and Dissemination

This study has been approved by the Health Research Authority and London – Hampstead Research Ethics Committee as application 19/LO/0181. The study findings will be published in peer-reviewed journals and presented at both conferences and stakeholder events.

For peer review only - http://bmjopen.bmj.com/site/about/guidelines.xhtml

References

1. Chen M, Fuqua J, Eugster EA. Characteristics of Referrals for Gender Dysphoria Over a 13-Year Period. J Adolesc Health. 2016 Mar 1;58(3):369–71.
2. Kaltiala R, Bergman H, Carmichael P, de Graaf NM, Egebjerg Rischel K, Frisén L, et al. Time trends in referrals to child and adolescent gender identity services: a study in four Nordic countries and in the UK. Nord J Psychiatry. 2020 Jan 2;74(1):40–4.
3. Referrals to GIDS, 2014-15 to 2018-19 [Internet]. Available from: https://gids.nhs.uk/number-referrals
4. Steensma TD, Cohen-Kettenis PT, Zucker KJ. Evidence for a Change in the Sex Ratio of Children Referred for Gender Dysphoria: Data from the Center of Expertise on Gender Dysphoria in Amsterdam (1988–2016). J Sex Marital Ther. 2018 Oct 3;44(7):713–5.
5. Aitken M, Steensma TD, Blanchard R, VanderLaan DP, Wood H, Fuentes A, et al. Evidence for an Altered Sex Ratio in Clinic-Referred Adolescents with Gender Dysphoria. J Sex Med. 2015;12(3):756–63.
6. Arnoldussen M, Steensma TD, Popma A, van der Miesen AIR, Twisk JWR, de Vries ALC. Re-evaluation of the Dutch approach: are recently referred transgender youth different compared to earlier referrals? Eur Child Adolesc Psychiatry [Internet]. 2019 Aug 31 [cited 2020 May 20]; Available from: https://doi.org/10.1007/s00787-019-01394-6
7. Achenbach, TM. The Achenbach system of empirically based assessemnt (ASEBA): development, findings, theory, and applications. Burlington, VT: University of Vermont Research Center for Children, Youth and Families; 2009.
8. Constantino, J. N., & Gruber, C. P. Social Responsiveness Scale (SRS). Los Angeles, CA: Western Psychological Services; 2005.

For peer review only

9. de Graaf NM, Cohen-Kettenis PT, Carmichael P, de Vries ALC, Dhondt K, Laridaen J, et al. Psychological functioning in adolescents referred to specialist gender identity clinics across Europe: a clinical comparison study between four clinics. Eur Child Adolesc Psychiatry. 2018 Jul 1;27(7):909–19.

10. Steensma TD, Cohen-Kettenis PT. Gender Transitioning before Puberty? Arch Sex Behav. 2011 Aug 1;40(4):649–50.

11. Olson KR, Durwood L, DeMeules M, McLaughlin KA. Mental Health of Transgender Children Who Are Supported in Their Identities. Pediatrics [Internet]. 2016 Mar 1 [cited 2020 Jun 23];137(3). Available from: https://pediatrics.aappublications.org/content/137/3/e20153223

12. Durwood L, McLaughlin KA, Olson KR. Mental Health and Self-Worth in Socially Transitioned Transgender Youth. J Am Acad Child Adolesc Psychiatry. 2017 Feb 1;56(2):116-123.e2.

13. Wong W, Drake SJ. A qualitative study of transgender children with early social transition: Parent perspectives and clinical implications. PEOPLE Int J Soc Sci. 2017 Nov 1;3(2).

14. Olson-Kennedy J, Cohen-Kettenis PT, Kreukels BPC, Meyer-Bahlburg HFL, Garofalo R, Meyer W, et al. Research Priorities for Gender Nonconforming/Transgender Youth: Gender Identity Development and Biopsychosocial Outcomes. Curr Opin Endocrinol Diabetes Obes. 2016 Apr;23(2):172–9.

15. Olson-Kennedy J, Chan Y-M, Garofalo R, Spack N, Chen D, Clark L, et al. Impact of Early Medical Treatment for Transgender Youth: Protocol for the Longitudinal, Observational Trans Youth Care Study. JMIR Res Protoc. 2019;8(7):e14434.

For peer review only

LOGIC retrospective analysis protocol

16. Tollit MA, Pace CC, Telfer M, Hoq M, Bryson J, Fulkoski N, et al. What are the health outcomes of trans and gender diverse young people in Australia? Study protocol for the Trans20 longitudinal cohort study. BMJ Open. 2019 Nov 1;9(11):e032151.
17. Spack NP, Edwards-Leeper L, Feldman HA, Leibowitz S, Mandel F, Diamond DA, et al. Children and Adolescents With Gender Identity Disorder Referred to a Pediatric Medical Center. Pediatrics. 2012 Mar 1;129(3):418–25.
18. Olson J, Schrager SM, Belzer M, Simons LK, Clark LF. Baseline Physiologic and Psychosocial Characteristics of Transgender Youth Seeking Care for Gender Dysphoria. J Adolesc Health. 2015 Oct 1;57(4):374–80.
19. de Vries ALC, Noens ILJ, Cohen-Kettenis PT, van Berckelaer-Onnes IA, Doreleijers TA. Autism Spectrum Disorders in Gender Dysphoric Children and Adolescents. J Autism Dev Disord. 2010 Aug 1;40(8):930–6.
20. Skagerberg E, Di Ceglie D, Carmichael P. Brief Report: Autistic Features in Children and Adolescents with Gender Dysphoria. J Autism Dev Disord. 2015 Aug 1;45(8):2628–32.
21. Longitudinal Outcomes of Gender Identity in Children [Internet]. The LOGIC study. [cited 2020 Apr 22]. Available from: https://logicstudy.uk/
22. Curtis LA, Burns A. Unit Costs of Health and Social Care 2019 [Internet]. Kent, UK: PSSRU; 2019 [cited 2020 Oct 5]. 176 p. Available from: https://doi.org/10.22024/UniKent/01.02.79286

Authors' contributions: EK, PC, RO, RH, RS, GB, SB-C, NdeG, TDS, AdeV, BY & MK contributed to the conception and design of the protocol. EK is the chief investigator of the LOGIC study. EK, CL, HS, VR & LS drafted the manuscript. RO & VV provided expertise

For peer review only

on statistical analysis. RH provided expertise on health economic analysis. All authors drafted or critically revised the protocol and approved the final version of the manuscript.

Funding statement: This work was supported by the National Institute for Health Research, Health Services and Delivery Research grant number 17/51/19.

Competing interests: The authors declare no competing interests.

Word Count: 2598 (excluding abstract, table and references)

For peer review only

LOGIC retrospective analysis protocol

Table 1: List of variables to be extracted for analysis for CYP aged 0-13 years who attended specialist gender services in the UK and the Netherlands between 2009 and 2017

Variable	Data Source	Level of data	Values
Demographic			
Age at referral	Medical record	Scale	Age in years and months
Age attended first appointment	Medical record	Scale	Age in years and months
Ethnicity*	Medical record	Nominal	1: White<br>2: Mixed<br>3: Asian or Asian British<br>4: Black or Black British<br>5: Chinese or other<br>6: Prefer not to say
Registered sex assignation at birth	Medical record	Nominal	1: Female<br>2: Male
Travel distance to gender identity clinic*	Medical record	Scale	Distance in kilometers
Family composition 1: Living situation	Medical record	Nominal	1: CYP Lives with both biological parents<br>2: CYP lives with one biological parent<br>3: Other

For peer review only - http://bmjopen.bmj.com/site/about/guidelines.xhtml

LOGIC retrospective analysis protocol

Family composition 2: LGBT parent	Medical record	Nominal	1: No<br>2: Yes
Family composition 3: CYP adopted	Medical record	Nominal	1: No<br>2: Yes
Primary caregiver age and relationship to CYP at referral	Medical record	Scale, Nominal	1: Mother<br>2: Father<br>3: Step-mother<br>4: Step-father<br>5: Adoptive mother<br>6: Adoptive father<br>7: Foster mother<br>8: Foster father<br>9: Foster parent (unspecified)<br>10: Aunt<br>11: Uncle<br>12: Grandparent
Sibling(s) age and sex*	Medical record	Scale, Nominal	1: Female<br>2: Male<br>3: Other<br>4: No sibling
Diagnostic			
Gender Dysphoria diagnosis	Medical record	Nominal	1: No<br>2: Yes
Gender Identity			
Current gender identity*	Medical record; Gender Identity Interview	Nominal	1: Female<br>2: Male<br>3: Non-binary

For peer review only

LOGIC retrospective analysis protocol

Social transition prior to first appointment	Medical record	Nominal	1: No<br>2: Yes<br>3: Partial
Social transition (at any time)*	Medical record	Nominal	1: No<br>2: Yes<br>3: Partial
Emotional and Behavioural Functioning			
Total problems	Child Behaviour Checklist (CBCL);	Ordinal	N/A
Internalising difficulties	Youth Self Report (YSR); Teacher	Ordinal	N/A
Externalising difficulties	Report Form (TRF)	Ordinal	N/A
Autism			
Autistic traits	Social Responsiveness Scale (SRS)	Ordinal	N/A
Referral Information			
Date of referral	Medical record	Scale	N/A

For peer review only

LOGIC retrospective analysis protocol

Date of first and last appointments	Medical record	Scale	N/A
Total number of appointments	Medical record	Scale	N/A
Date of discharge*	Medical record	Scale	N/A
Reason for discharge*	Medical record	Nominal	1: Discharged against professional advice<br>2: Discharged on professional advice<br>3: Inappropriate Referral<br>4: Patient moved out of the area<br>5: Patient non-attendance<br>6: Patient requested discharge<br>7: Transferred from CAMHS to local Adult Mental Health Services<br>8: Transferred to other Health Care Provider not Medium/High Secure<br>98: Patient not yet discharged
Healthcare Resource Use			
Type of clinical appointments	Medical record	Nominal	T&P NHSFT:<br>1: Early Liaison with endocrine clinic for <15 yrs[a]<br>2: Liaison with endocrine clinic for 15+ yrs[a]<br>3: GIDS Outreach Assessment[b]<br>4: GIDS Standard Assessment[b]<br>5: GIDS Treatment Outreach[c]<br>6: GIDS Treatment Standard[c]<br>7: GIDS Young Persons Group[d]<br>8: Group (not specified)[d]<br>9: GIDS Transitions Appointment[c]<br>10: Endocrinology (15+ yrs)[a]<br>11: Endocrinology (<15 yrs)[a]

For peer review only

LOGIC retrospective analysis protocol

			12: Child, Young Adults and Families (CYAF) Assessment[b]<br>13: CYAF Individual therapy once per week[c]<br>14: CYAF Family Therapy[c]<br><br>AUMC:<br>1: Intake Psychology[b]<br>2: Consultation Psychology[b]<br>3: Psychological Assessment[b]<br>4: Screening Psychiatry[b]<br>5: Consultation Psychiatry[b]<br>6: New patient endocrinology[a]<br>7: Consultation endocrinology[a]<br>8: Group consultation endocrinology[a]<br>9: DEXA scan[a]<br>10: Labs[a]<br>11: Consultation fertility[a]
Date of appointments/frequency	Medical record	Scale	N/A
Duration of appointments	Medical record	Scale	Time in minutes
Patient attendance	Medical record	Nominal	1: Attended<br>2: Cancelled<br>3: Did not attend
Clinician attendance**	Medical record	Nominal	1: Psychologist<br>2: Psychologist assistant<br>3: Psychiatrist<br>4: Endocrinologist<br>5: Doctors assistant<br>6: Nurse

For peer review only

For peer review only - http://bmjopen.bmj.com/site/about/guidelines.xhtml

	7: Gynaecologist<br>8: Sexologist<br>9: Doctor<br>10: Unknown

*Data available for T&P NHSFT only
**Data available for AUMC only

Note. Appointment type groupings: [a]Endocrinology/Medical; [b]Assessment; [c]Psychosocial Treatment; [d]Group

For peer review only

Appendix 1. Information regarding care pathways

In the UK, following the NHS England service specification, all patients attend the service for assessment in the first instance. According to the service specifications, this typically consists of 3-6 appointments, with the number of appointments based on individual need, agreed with the young person and their family. At the end of the assessment, if the young person remains in contact with the service they either continue to explore their gender identity and options around this, or they may be referred to the endocrine clinic with ongoing exploration of gender and pathways and psychosocial support. The current NHS service specification can be found here: https://www.england.nhs.uk/wp-content/uploads/2017/04/gender-development-service-children-adolescents.pdf. Please note that this is currently undergoing a scheduled review, and an amendment was made to the service specification in December 2020 following the Bell vs. Tavistock judicial review. The amendment can be found here: https://www.england.nhs.uk/wp-content/uploads/2020/12/Amendment-to-Gender-Identity-Development-Service-Specification-for-Children-and-Adolescents.pdf

As GIDS is a national service, 'outreach' assessment simply refers to the assessment being undertaken remotely from the main clinic base in London. After an assessment, if the CYP remains in contact with the service, appointment type then changes to 'treatment': 'treatment outreach' would indicate the treatment took place outside the main London clinic base, whereas 'treatment standard' would mean that the treatment took place at the London clinic. We will certainly provide additional descriptions when reporting findings.

In the Netherlands, the first consultation ('intake') is always with a child and adolescent psychiatrist. The following appointments during the assessment phase are with a psychologist. This usually consists of 3-6 appointments (on a monthly basis), with the number of appointments based on individual need, and agreed with the young person and

For peer review only

their family. At the end of the assessment, the majority of CYP are referred to endocrinology and also continue to see a psychologist for psychosocial support. The young person and their family are seen by the endocrinologist and psychologist every 3 months.

For peer review only

Appendix 2. Information regarding appointment types in the specialist gender clinics

	Type of clinical appointment	Definition
T&P NHSFT		
	1: Early Liaison with endocrine clinic for <15 yrs[a]	Appointment with the endocrinology clinic for those aged 15+ years
	2: Liaison with endocrine clinic for 15+ yrs[a]	Appointment with the endocrinology clinic for those aged <15 years
	3: GIDS Outreach Assessment[b]	Assessment undertaken remotely from the main clinic base in London
	4: GIDS Standard Assessment[b]	Assessment which took place at the London clinic
	5: GIDS Treatment Outreach[c]	Psychosocial treatment undertaken remotely from the main clinic base in London
	6: GIDS Treatment Standard[c]	Psychosocial treatment which took place at the London clinic
	7: GIDS Young Persons Group[d]	Group appointment specifically for young people (take place during GIDS group sessions)
	8: Group (not specified)[d]	Group appointment (take place during GIDS group sessions)
	9: GIDS Transitions Appointment[c]	Appointment for young people with GIDS and GIC clinician to discuss transition to adult service
	10: Endocrinology (15+ yrs)$_a$	Appointment with the endocrinology clinic for those aged 15+ years
	11: Endocrinology (<15 yrs)$_a$	Appointment with the endocrinology clinic for those aged <15 years
	12: Child, Young Adults and Families (CYAF) Assessment[b]	Assessment by CYAF clinician
	13: CYAF Individual therapy once per week[c]	Individual therapy by a CYAF clinician
	14: CYAF Family Therapy[c]	Family therapy by a CYAF clinician
AUMC		
	1: Intake Psychology[b]	First GID appointment with the psychologist during the diagnostic phase, 60 min
	2: Consultation Psychology[b]	Standard GID assessment with the psychologist during the diagnostic phase, 60 min

For peer review only

3: Psychological Assessment[b]	Appointment for completing questionnaires & psychiatric assessment / interview during the diagnostic phase, 2-3h
4: Screening Psychiatry[b]	First appointment after referral to the AUMC is with the main practitioner / treatment provider<br>For children/adolescents the appointed main practitioner in the hospital is the Child and Adolescent psychiatrist<br>After the psychiatric screening, the psychiatrist refers the CYP to a psychologist for the GID assessment / diagnostic phase
5: Consultation Psychiatry[b]	Psychiatric assessment during the diagnostic phase<br>Usually upon request from psychologist (for example in case when CYP presents with (complex) psychiatric comorbidity)
6: New patient endocrinology[a]	First appointment with the endocrinology clinic for CYP
7: Consultation endocrinology[a]	Standard appointment with the endocrinology clinic for CYP
8: Group consultation endocrinology / Webinar[a]	Group consultation before start medical treatment (providing education/information re medical treatment)
9: DEXA scan[a]	Order for DEXA scan to check bone health
10: Labs[a]	Appointment with the nurse for bloods / weight / height measurements and standard health checks
11: Consultation fertility[a]	Appointment with the fertility doctor (GYN)

Note. Appointment type groupings: [a]Endocrinology/Medical; [b]Assessment; [c]Psychosocial Treatment; [d]Group

For peer review only

BMJ Open

Longitudinal Outcomes of Gender Identity in Children (LOGIC): study protocol for a retrospective analysis of the characteristics and outcomes of children referred to specialist gender services in the UK and the Netherlands

Journal:	*BMJ Open*
Manuscript ID	bmjopen-2021-054895.R1
Article Type:	Protocol
Date Submitted by the Author:	09-Aug-2021
Complete List of Authors:	Kennedy, Eilis; Tavistock and Portman NHS Foundation Trust, Research & Development; University College London, Research Department of Clinical, Educational and Health Psychology<br>Lane, Chloe; Tavistock and Portman NHS Foundation Trust, Research & Development<br>Stynes, Hannah; Tavistock and Portman NHS Foundation Trust<br>Ranieri, Veronica; Tavistock and Portman NHS Foundation Trust, Research & Development; University College London, Science & Technology Studies<br>Spinner, Lauren; Tavistock and Portman NHS Foundation Trust, Research & Development<br>Carmichael, Polly; Tavistock and Portman NHS Foundation Trust, Gender Identity Development Service<br>Omar, Rumana; University College London,<br>Vickerstaff, Victoria; University College London, UCL Mental Health Sciences Unit<br>Hunter, Rachael; University College London, Research Dept of Primary Care and Population Health<br>Senior, Robert; Tavistock and Portman NHS Foundation Trust<br>Butler, Gary; University College London Hospital, Paediatrics and Adolescents<br>Baron-Cohen, Simon; University of Cambridge<br>De Graaf, Nastasja; Amsterdam University Medical Centres<br>Steensma, Thomas; Amsterdam University Medical Centres, Center of Expertise on Gender Dysphoria<br>de Vries, Annelou; VU Medisch Centrum, Child and Adolescent Psychiatry<br>Young, Bridget; University of Liverpool<br>King, Michael; University College Medical School, Division of Psychiatry
**Primary Subject Heading**:	Mental health
Secondary Subject Heading:	Health services research
Keywords:	Child & adolescent psychiatry < PSYCHIATRY, Sexual and gender disorders < PSYCHIATRY, Health economics < HEALTH SERVICES ADMINISTRATION & MANAGEMENT

For peer review only - http://bmjopen.bmj.com/site/about/guidelines.xhtml

SCHOLARONE™
Manuscripts

I, the Submitting Author has the right to grant and does grant on behalf of all authors of the Work (as defined in the below author licence), an exclusive licence and/or a non-exclusive licence for contributions from authors who are: i) UK Crown employees; ii) where BMJ has agreed a CC-BY licence shall apply, and/or iii) in accordance with the terms applicable for US Federal Government officers or employees acting as part of their official duties; on a worldwide, perpetual, irrevocable, royalty-free basis to BMJ Publishing Group Ltd ("BMJ") its licensees and where the relevant Journal is co-owned by BMJ to the co-owners of the Journal, to publish the Work in this journal and any other BMJ products and to exploit all rights, as set out in our licence.

The Submitting Author accepts and understands that any supply made under these terms is made by BMJ to the Submitting Author unless you are acting as an employee on behalf of your employer or a postgraduate student of an affiliated institution which is paying any applicable article publishing charge ("APC") for Open Access articles. Where the Submitting Author wishes to make the Work available on an Open Access basis (and intends to pay the relevant APC), the terms of reuse of such Open Access shall be governed by a Creative Commons licence – details of these licences and which Creative Commons licence will apply to this Work are set out in our licence referred to above.

Other than as permitted in any relevant BMJ Author's Self Archiving Policies, I confirm this Work has not been accepted for publication elsewhere, is not being considered for publication elsewhere and does not duplicate material already published. I confirm all authors consent to publication of this Work and authorise the granting of this licence.

For peer review only

Longitudinal Outcomes of Gender Identity in Children (LOGIC): study protocol for a retrospective analysis of the characteristics and outcomes of children referred to specialist gender services in the UK and the Netherlands

Eilis Kennedy[1,2]; Chloe Lane[1]; Hannah Stynes[1]; Veronica Ranieri[1]; Lauren Spinner[1]; Polly Carmichael[3]; Rumana Omar[4]; Victoria Vickerstaff[5,12]; Rachael Hunter[5]; Rob Senior[1]; Gary Butler[3,6]; Simon Baron-Cohen[7]; Nastasja M. de Graaf[8,9]; Thomas D. Steensma[8,9]; Annelou L.C. de Vries[8,10]; Bridget Young[11]; Michael King[12]

[1] Research & Development Unit, Tavistock Centre, Tavistock and Portman NHS Foundation Trust

[2] Research Department of Clinical, Educational and Health Psychology, University College London

[3] Gender Identity Development Service, Tavistock and Portman NHS Foundation Trust

[4] Department of Statistical Science, University College London

[5] Research Department of Primary Care and Population Health, Royal Free Medical School, University College London

[6] Department of Paediatric and Adolescent Endocrinology, University College London Hospital and University College London, Great Ormond Street Institute of Child Health

[7] Autism Research Centre, Department of Psychiatry, University of Cambridge

[8] Center of Expertise on Gender Dysphoria, Amsterdam University Medical Centres

For peer review only

LOGIC retrospective analysis protocol

[9] Department of Medical Psychology, Amsterdam University Medical Center

[10] Department of Child and Adolescent Psychiatry, Amsterdam University Medical Center

[11] Institute of Population Health Sciences, University of Liverpool

[12] Division of Psychiatry, Faculty of Brain Sciences, University College London

Address for correspondence: Dr Eilis Kennedy, Research & Development Unit, Tavistock Centre, Tavistock & Portman NHS Foundation Trust, 120 Belsize Lane, NW3 5AS, United Kingdom (e-mail: ekennedy@tavi-port.nhs.uk).

For peer review only

LOGIC retrospective analysis protocol

Abstract

Introduction

Specialist gender services for children and young people (CYP) worldwide have experienced a significant increase in referrals in recent years. As rates of referrals increase, it is important to understand the characteristics and profile of CYP attending these services in order to inform treatment pathways and to ensure optimal outcomes.

Methods and Analysis

A retrospective observational study of clinical health records from specialist gender services for CYP in the UK and the Netherlands. The retrospective analysis will examine routinely collected clinical and outcome measures data including demographic, clinical, gender identity-related, and healthcare resource use information. Data will be reported for each service and also compared between services. This study forms part of a wider programme of research investigating outcomes of gender identity in children (the LOGIC study).

Ethics and Dissemination

The proposed study has been approved by the UK Health Research Authority and London – Hampstead Research Ethics Committee as application 19/LO/0181. The study findings will be published in peer-reviewed journals and presented at both conferences and stakeholder events.

For peer review only

Article Summary

Strengths and limitations

- This study will involve a retrospective analysis of routinely collected data from two European specialist gender services in a large cohort of CYP aged ≤13 years.
- A detailed evaluation of service use and costs will be ascertained for specialist gender services for CYP in both the UK and the Netherlands.
- As data from two distinct specialist gender services (in the UK and the Netherlands) will be utilised, not all variables will be available for CYP from both services.
- As the study will use data extracted from clinical health records, there will inevitably be some missing data but this will be taken into account in the analyses.
- Selection of measures and variables is constrained to those that are routinely collected by the services but limitations of these will be discussed when interpreting the findings.

For peer review only

Introduction

In recent years, specialist gender services for CYP have experienced a significant rise in referrals worldwide (1,2). For example, the gender identity development service (GIDS) at the Tavistock & Portman NHS Foundation Trust (NHSFT) in the UK has reported a 382% increase from 678 referrals in 2014/15 to 2,590 referrals in 2018/19 (3). Time trends in relation to the profile of CYP referred to services have also been noted. In particular, there has been a shift in recent years to an increase in referrals of CYP assigned female at birth (4,5). However, a recent study of time trends in adolescent referrals in the Netherlands found that, other than a shift in sex ratio, no other time trends were observed in relation to demographics or intensity of gender dysphoria (6). As rates of referrals increase, it is important to understand the characteristics and profile of CYP attending these services in order to inform treatment pathways and to ensure optimal outcomes for these CYP.

The specialist gender services at the Tavistock & Portman NHSFT in the UK and Amsterdam University Medical Centre (AUMC) in the Netherlands are two of the longest established and largest services in Europe for CYP seeking support in relation to their gender identity. Both of the services follow a similar assessment protocol and both have consistently used the same outcome measures over the past 8 years, enabling comparisons to be made between the services. These include a multi-disciplinary clinical assessment and completion of the Child Behaviour Checklist (CBCL), the Youth Self Report (YSR) and the Teacher Report Form (TRF) (7) to assess emotional and behavioural functioning, and the Social Responsiveness Scale (SRS) (8) to assess autistic traits. Data from these services have previously been compared and some cross-cultural differences in the nature of referrals to

For peer review only - http://bmjopen.bmj.com/site/about/guidelines.xhtml

these clinics have previously been reported. For example, a recent study comparing psychological functioning in adolescents (aged 12-18 years) referred to specialist gender services across four European countries (including the UK and the Netherlands) identified that, at the time of referral, emotional and behavioural problems and peer relationship difficulties were most prevalent in CYP presenting to services in the UK (9). Conversely, these issues were least prevalent in CYP presenting to services in the Netherlands. In addition, a greater number of younger children presenting to specialist gender services who have already made changes in their clothing, hairstyle, first name and pronouns to reflect their gender identity (sometimes referred to as social transition) at the time of referral has been reported(10). Recent research has suggested that making these changes early on can have desirable outcomes for CYP (11–13). However, research on this matter in pre-pubertal children is limited, and thus we need to know more about how making such changes in dress and behaviour relates to later outcomes (14). This study will extend current understanding of the nature of CYP referrals, particularly in relation to younger children, by providing further opportunity to characterise the profile and outcomes of attendees at each service, whilst also exploring potential cross-cultural differences.

Several longitudinal prospective cohort studies of CYP attending specialist gender services are now ongoing (15,16). However, retrospective analysis of clinical data can provide important and unique insights into the characteristics and outcomes of CYP referred to these services which are not yet available from these ongoing prospective studies. This is particularly pertinent as it is widely acknowledged that the evidence base on which current treatment protocols is based is limited (14). Furthermore, retrospective studies include a whole specified cohort, which is not necessarily feasible within prospective studies which generally require a process of recruitment and novel data collection. Retrospective studies of clinical cohorts therefore provide a valuable and informative addition to the literature.

For peer review only - http://bmjopen.bmj.com/site/about/guidelines.xhtml

As much of the existing literature has focused on adolescents, little is currently known about the overall characteristics and outcomes of younger CYP, particularly pre-pubertal and early pubertal children, who attend specialist gender services. Consequently, there is limited evidence to inform the likely trajectories and outcomes of these CYP and to enable clinical care pathways to be tailored accordingly. The present study aims to address this gap in the literature by profiling CYP aged ≤13 years who first attended specialist gender services across an eight year period (2009-2017). It will describe and, where possible, compare the outcomes of CYP attendees in relation to their demographic and family backgrounds, emotional and behavioural functioning, autistic traits and gender identity (e.g. diagnosis of gender dysphoria and social transition). Measures relating to emotional and behavioural functioning and autistic traits will be included, as mental health conditions and autism have been reported to co-occur for some CYP who are referred to specialist gender services (17–20). Differences in referrals and treatment pathways in each country will be explored e.g. numbers of children presenting to the clinic who have already socially transitioned and age at time of referral to paediatric endocrinology. Service use and outcomes will be identified, particularly in relation to CYP who attend a paediatric endocrinology clinic and those who do not. A healthcare resource costing of both services will also be undertaken. It is anticipated that the proposed research will improve understanding of the characteristics of service users in order to help in the planning and organisation of services and to address the need for tailored support when required.

Aims

This study aims to identify 1) the profile of CYP aged ≤13 years attending specialist gender services in the UK and the Netherlands between 2009 and 2017; 2) the proportion of these CYP who a) experience gender dysphoria, b) socially transition, c) access medical treatment (e.g. hormone blockers and cross sex hormones) and d) have co-occurring autistic

For peer review only - http://bmjopen.bmj.com/site/about/guidelines.xhtml

traits; 3) the profile of CYP who attend paediatric endocrinology clinics and the profile of CYP who do not attend these clinics; 4) the service use and costs of CYP attending specialist gender services; 5) costs for CYP who attend paediatric endocrinology clinics and costs for CYP who do not attend these clinics.

Methods and Analysis

Study design

A retrospective observational study of clinical health records from specialist gender services in the UK and the Netherlands across an eight year period (2009-2017). See Table 1 for a full list of variables and measures. This study forms part of a wider programme of research investigating outcomes of gender identity in children (the LOGIC study) (21). This programme of research utilises a mixed methods approach, incorporating both quantitative and qualitative longitudinal studies to investigate the experiences, outcomes and wellbeing of families referred to the UK GIDS.

[Insert Table 1 about here]

Study population

The study population will consist of all CYP aged ≤13 years who attended at least one appointment at a specialist gender service (GIDS or AUMC) between 2009 and 2017 and were recorded in the electronic patient records system used by the services. This will include approximately 1040 CYP from GIDS and 529 CYP from AUMC. CYP with differences in sex development and those referred to the service to obtain support with a parent undergoing gender transition will be excluded from the analyses.

For peer review only

LOGIC retrospective analysis protocol

Data source

The study will consist of a retrospective analysis of routinely collected clinical data extracted from both the Tavistock & Portman NHSFT and the AUMC in the Netherlands between 2009 and 2017. The UK GIDS was established in 1989 and is currently one of the largest, if not the largest, specialist gender clinic for CYP in the world. It is a nationally commissioned service covering England, Wales, Northern Ireland and in part, Scotland and the Republic of Ireland, through a series of outreach clinics and a main hub in London. The Center of Expertise on Gender Dysphoria in the Netherlands was established in 1988, and is one of the oldest and most established clinics. These two sites were chosen as they represent two of the largest and longest serving specialist clinics in Europe for CYP seeking support relating to their gender identity. These services are therefore uniquely placed to undertake a retrospective analysis of the characteristics and outcomes of CYP who attended the clinics across an eight year period. Completion of assessment and outcome measures such as the CBCL, YSR, TRF, and SRS was entirely voluntary and not a condition of receiving care. The data from these measures were collected retrospectively for this study.

Procedure

The research teams at each respective site (the Tavistock & Portman NHSFT in the UK and AUMC) will submit a request for data extraction to their local informatics team. Data will be extracted by each local informatics team via their electronic service user record software and entered into a CSV-formatted dataset. Members of the research teams at each respective site will manually input any data into the datasets which cannot be extracted by the informatics teams, such as handwritten or typed information obtained from assessment reports. Data for social transition will be hand-searched by the UK research team from medical records, although the content of which can vary enormously from patient-to-patient and by clinician. All identifiable data will be held in a password-protected database on an

For peer review only

encrypted NHS server at the Tavistock and Portman NHSFT until the dataset has been finalised and is ready for analysis. Once the dataset is complete, all identifiable data (NHS patient IDs, dates of birth and all free-text responses) will be removed. The anonymised dataset will then be uploaded and stored onto an encrypted and GDPR-compliant data portal (Data Safe Haven) so that the statistical team at University College London (UCL) PRIMENT's Clinical Trials Unit can access the dataset for analysis. A data sharing agreement will be in place between the Tavistock & Portman NHSFT and the AUMC, each as data controllers. The sites will share ownership of the anonymised datasets once analysis is complete and these datasets will be retained for no longer than 20 years. A collaboration agreement will also be enforced between all participating sites, identifying UCL as the data processor. The data processor shall destroy the data upon request by the Tavistock & Portman NHSFT. The study will run for approximately 2 years (2019 – 2021).

Analysis plan

Data will be analysed in STATA by the statistical team at UCL PRIMENT's Clinical Trials Unit. Characteristics of the CYP will be described using mean (SD), median (IQ range) or frequencies (proportion), as appropriate. In order to address study aim (2), the proportion of CYP who experience gender dysphoria, socially transition, access physical/medical treatment (i.e. attend a paediatric endocrinology clinic) and have co-occurring autistic traits (as measured by the SRS) will be estimated, along with their 95% confidence intervals. In order to address study aim (3), descriptive characteristics of the CYP who attend a paediatric endocrinology clinic will be compared to those for CYP who do not attend such a clinic. Regression models will be used to examine factors that are associated with referral to a paediatric endocrinology clinic. Factors which are likely to be explored within these models include: a) family composition, b) social transition, c) emotional and behavioural functioning

For peer review only

of CYP (measured by CBCL, YSR and TRF) and d) autistic traits (measured by the SRS). Regression analyses will be adjusted for year of referral.

In relation to study aim (4), the average number of contacts with the service will be reported for each type of appointment. To establish healthcare resource costs, data pertaining to individual appointments (i.e. assessment appointment; psychosocial treatment appointment; group appointment; and endocrine appointment) from the time of referral until discharge from the service will be analysed. These will be costed based on information provided by the service on the number, profession and grade of clinical involvement for each appointment type and using information from the Personal Social Service Resource Unit (PSSRU) to calculate cost per minute (22). The cost per minute for each appointment type will then be multiplied by the duration of appointment, as recorded in patient files. We will conduct a sensitivity analysis using only service provided costs and only NHS Reference Costs (22). The Netherlands will be costed based on PSSRU costing, with a sensitivity analysis using Netherlands specific wages. Costed clinic appointments will then be summed together to calculate the total cost of care for each CYP and divided by contact time to adjust for patients with longer follow-ups. Average total costs of care and appointments will be reported for young people who attended a paediatric endocrinology clinic versus those who did not. These will be reported separately for the UK and the Netherlands. Further information regarding appointment types is provided in Appendix 1.

In order to address study aim (5), information such as (i) costs of care; (ii) country of care; (iii) care pathway (the type of treatment and/or support that the CYP receives throughout their time with the service); (iv) outcomes including wellbeing (measured by CBCL, YSR and TRF); and (v) potential predictors of costs and outcomes (e.g. age at first appointment, gender dysphoria diagnosis and autistic traits) will be used to explore differences in costs between CYP who attend a paediatric endocrinology clinic and those who

For peer review only

do not. The purpose of this analysis is to calculate the cost of care for each CYP from the beginning to end of their time with GIDS, and then report if different CYP have different costs because (i) of the country they are in; (ii) they followed the endocrine pathway (or didn't); (iii) other clinical factors such as the prevalence of autistic traits. Further information regarding care pathways is described in Appendix 2.

All of the analyses will be presented individually for each service and also combined where possible. Where appropriate, analyses will be reported by year (2009 to 2017). If necessary, previously identified differences in baseline presentation of CYP referrals to the two services will be taken into account in the analyses, as well as other differences between the clinics such as the time (year) at which early physical interventions are offered. Potential bias due to missing data will be investigated by comparing the characteristics of CYP who have completed the reported outcome measures to those who have incomplete or no outcome data. Outcome measure data (i.e. CBCL, TRF, YSR, and SRS) will only be included in analyses when ≥70% of the cohort have completed the measures.

Patient and public involvement statement

The LOGIC study was developed in collaboration with UK GIDS users. The research proposal was also discussed at a stakeholder event involving trans youth organisations. The LOGIC study has a patient and public involvement (PPI) group, comprised of parents and CYP who are participating in our ongoing longitudinal cohort study. Findings and outputs will be discussed with the study PPI group.

Ethics and Dissemination

This study has been approved by the Health Research Authority and London – Hampstead Research Ethics Committee as application 19/LO/0181. The study findings will

For peer review only - http://bmjopen.bmj.com/site/about/guidelines.xhtml

LOGIC retrospective analysis protocol

be published in peer-reviewed journals and presented at both conferences and stakeholder events.

References

1. Chen M, Fuqua J, Eugster EA. Characteristics of Referrals for Gender Dysphoria Over a 13-Year Period. J Adolesc Health. 2016 Mar 1;58(3):369–71.
2. Kaltiala R, Bergman H, Carmichael P, de Graaf NM, Egebjerg Rischel K, Frisén L, et al. Time trends in referrals to child and adolescent gender identity services: a study in four Nordic countries and in the UK. Nord J Psychiatry. 2020 Jan 2;74(1):40–4.
3. Referrals to GIDS, 2014-15 to 2018-19 [Internet]. Available from: https://gids.nhs.uk/number-referrals
4. Steensma TD, Cohen-Kettenis PT, Zucker KJ. Evidence for a Change in the Sex Ratio of Children Referred for Gender Dysphoria: Data from the Center of Expertise on Gender Dysphoria in Amsterdam (1988–2016). J Sex Marital Ther. 2018 Oct 3;44(7):713–5.
5. Aitken M, Steensma TD, Blanchard R, VanderLaan DP, Wood H, Fuentes A, et al. Evidence for an Altered Sex Ratio in Clinic-Referred Adolescents with Gender Dysphoria. J Sex Med. 2015;12(3):756–63.
6. Arnoldussen M, Steensma TD, Popma A, van der Miesen AIR, Twisk JWR, de Vries ALC. Re-evaluation of the Dutch approach: are recently referred transgender youth different compared to earlier referrals? Eur Child Adolesc Psychiatry [Internet]. 2019 Aug 31 [cited 2020 May 20]; Available from: https://doi.org/10.1007/s00787-019-01394-6
7. Achenbach, TM. The Achenbach system of empirically based assessemnt (ASEBA): development, findings, theory, and applications. Burlington, VT: University of Vermont Research Center for Children, Youth and Families; 2009.

For peer review only

8. Constantino, J. N., & Gruber, C. P. Social Responsiveness Scale (SRS). Los Angeles, CA: Western Psychological Services; 2005.

9. de Graaf NM, Cohen-Kettenis PT, Carmichael P, de Vries ALC, Dhondt K, Laridaen J, et al. Psychological functioning in adolescents referred to specialist gender identity clinics across Europe: a clinical comparison study between four clinics. Eur Child Adolesc Psychiatry. 2018 Jul 1;27(7):909–19.

10. Steensma TD, Cohen-Kettenis PT. Gender Transitioning before Puberty? Arch Sex Behav. 2011 Aug 1;40(4):649–50.

11. Olson KR, Durwood L, DeMeules M, McLaughlin KA. Mental Health of Transgender Children Who Are Supported in Their Identities. Pediatrics [Internet]. 2016 Mar 1 [cited 2020 Jun 23];137(3). Available from: https://pediatrics.aappublications.org/content/137/3/e20153223

12. Durwood L, McLaughlin KA, Olson KR. Mental Health and Self-Worth in Socially Transitioned Transgender Youth. J Am Acad Child Adolesc Psychiatry. 2017 Feb 1;56(2):116-123.e2.

13. Wong W, Drake SJ. A qualitative study of transgender children with early social transition: Parent perspectives and clinical implications. PEOPLE Int J Soc Sci. 2017 Nov 1;3(2).

14. Olson-Kennedy J, Cohen-Kettenis PT, Kreukels BPC, Meyer-Bahlburg HFL, Garofalo R, Meyer W, et al. Research Priorities for Gender Nonconforming/Transgender Youth: Gender Identity Development and Biopsychosocial Outcomes. Curr Opin Endocrinol Diabetes Obes. 2016 Apr;23(2):172–9.

15. Olson-Kennedy J, Chan Y-M, Garofalo R, Spack N, Chen D, Clark L, et al. Impact of Early Medical Treatment for Transgender Youth: Protocol for the Longitudinal, Observational Trans Youth Care Study. JMIR Res Protoc. 2019;8(7):e14434.

For peer review only

LOGIC retrospective analysis protocol

16. Tollit MA, Pace CC, Telfer M, Hoq M, Bryson J, Fulkoski N, et al. What are the health outcomes of trans and gender diverse young people in Australia? Study protocol for the Trans20 longitudinal cohort study. BMJ Open. 2019 Nov 1;9(11):e032151.
17. Spack NP, Edwards-Leeper L, Feldman HA, Leibowitz S, Mandel F, Diamond DA, et al. Children and Adolescents With Gender Identity Disorder Referred to a Pediatric Medical Center. Pediatrics. 2012 Mar 1;129(3):418–25.
18. Olson J, Schrager SM, Belzer M, Simons LK, Clark LF. Baseline Physiologic and Psychosocial Characteristics of Transgender Youth Seeking Care for Gender Dysphoria. J Adolesc Health. 2015 Oct 1;57(4):374–80.
19. de Vries ALC, Noens ILJ, Cohen-Kettenis PT, van Berckelaer-Onnes IA, Doreleijers TA. Autism Spectrum Disorders in Gender Dysphoric Children and Adolescents. J Autism Dev Disord. 2010 Aug 1;40(8):930–6.
20. Skagerberg E, Di Ceglie D, Carmichael P. Brief Report: Autistic Features in Children and Adolescents with Gender Dysphoria. J Autism Dev Disord. 2015 Aug 1;45(8):2628–32.
21. Longitudinal Outcomes of Gender Identity in Children [Internet]. The LOGIC study. [cited 2020 Apr 22]. Available from: https://logicstudy.uk/
22. Curtis LA, Burns A. Unit Costs of Health and Social Care 2019 [Internet]. Kent, UK: PSSRU; 2019 [cited 2020 Oct 5]. 176 p. Available from: https://doi.org/10.22024/UniKent/01.02.79286

Authors' contributions: EK, PC, RO, RH, RS, GB, SB-C, NdeG, TDS, AdeV, BY & MK contributed to the conception and design of the protocol. EK is the chief investigator of the LOGIC study. EK, CL, HS, VR & LS drafted the manuscript. RO & VV provided expertise

For peer review only

on statistical analysis. RH provided expertise on health economic analysis. All authors drafted or critically revised the protocol and approved the final version of the manuscript.

Funding statement: This work was supported by the National Institute for Health Research, Health Services and Delivery Research grant number 17/51/19.

Competing interests: The authors declare no competing interests.

Word Count: 2598 (excluding abstract, table and references)

For peer review only

LOGIC retrospective analysis protocol

Table 1: List of variables to be extracted for analysis for CYP aged 0-13 years who attended specialist gender services in the UK and the Netherlands between 2009 and 2017

Variable	Data Source	Level of data	Values
Demographic			
Age at referral	Medical record	Scale	Age in years and months
Age attended first appointment	Medical record	Scale	Age in years and months
Ethnicity*	Medical record	Nominal	1: White<br>2: Mixed<br>3: Asian or Asian British<br>4: Black or Black British<br>5: Chinese or other<br>6: Prefer not to say
Registered sex assignation at birth	Medical record	Nominal	1: Female<br>2: Male
Travel distance to gender identity clinic*	Medical record	Scale	Distance in kilometers
Family composition 1: Living situation	Medical record	Nominal	1: CYP Lives with both biological parents<br>2: CYP lives with one biological parent<br>3: Other

For peer review only

LOGIC retrospective analysis protocol

Family composition 2: LGBT parent	Medical record	Nominal	1: No<br>2: Yes
Family composition 3: CYP adopted	Medical record	Nominal	1: No<br>2: Yes
Primary caregiver age and relationship to CYP at referral	Medical record	Scale, Nominal	1: Mother<br>2: Father<br>3: Step-mother<br>4: Step-father<br>5: Adoptive mother<br>6: Adoptive father<br>7: Foster mother<br>8: Foster father<br>9: Foster parent (unspecified)<br>10: Aunt<br>11: Uncle<br>12: Grandparent
Sibling(s) age and sex*	Medical record	Scale, Nominal	1: Female<br>2: Male<br>3: Other<br>4: No sibling
Diagnostic			
Gender Dysphoria diagnosis	Medical record	Nominal	1: No<br>2: Yes
Gender Identity			
Current gender identity*	Medical record; Gender Identity Interview	Nominal	1: Female<br>2: Male<br>3: Non-binary

For peer review only - http://bmjopen.bmj.com/site/about/guidelines.xhtml

Social transition prior to first appointment	Medical record	Nominal	1: No<br>2: Yes<br>3: Partial
Social transition (at any time)*	Medical record	Nominal	1: No<br>2: Yes<br>3: Partial
Emotional and Behavioural Functioning			
Total problems	Child Behaviour Checklist (CBCL);	Ordinal	N/A
Internalising difficulties	Youth Self Report (YSR); Teacher	Ordinal	N/A
Externalising difficulties	Report Form (TRF)	Ordinal	N/A
Autism			
Autistic traits	Social Responsiveness Scale (SRS)	Ordinal	N/A
Referral Information			
Date of referral	Medical record	Scale	N/A

For peer review only - http://bmjopen.bmj.com/site/about/guidelines.xhtml

LOGIC retrospective analysis protocol

Date of first and last appointments	Medical record	Scale	N/A
Total number of appointments	Medical record	Scale	N/A
Date of discharge*	Medical record	Scale	N/A
Reason for discharge*	Medical record	Nominal	1: Discharged against professional advice<br>2: Discharged on professional advice<br>3: Inappropriate Referral<br>4: Patient moved out of the area<br>5: Patient non-attendance<br>6: Patient requested discharge<br>7: Transferred from CAMHS to local Adult Mental Health Services<br>8: Transferred to other Health Care Provider not Medium/High Secure<br>98: Patient not yet discharged
Healthcare Resource Use			
Type of clinical appointments	Medical record	Nominal	T&P NHSFT:<br>1: Early Liaison with endocrine clinic for <15 yrs[a]<br>2: Liaison with endocrine clinic for 15+ yrs[a]<br>3: GIDS Outreach Assessment[b]<br>4: GIDS Standard Assessment[b]<br>5: GIDS Treatment Outreach[c]<br>6: GIDS Treatment Standard[c]<br>7: GIDS Young Persons Group[d]<br>8: Group (not specified)[d]<br>9: GIDS Transitions Appointment[c]<br>10: Endocrinology (15+ yrs)[a]<br>11: Endocrinology (<15 yrs)[a]

For peer review only

LOGIC retrospective analysis protocol

			12: Child, Young Adults and Families (CYAF) Assessment[b]<br>13: CYAF Individual therapy once per week[c]<br>14: CYAF Family Therapy[c]<br><br>AUMC:<br>1: Intake Psychology[b]<br>2: Consultation Psychology[b]<br>3: Psychological Assessment[b]<br>4: Screening Psychiatry[b]<br>5: Consultation Psychiatry[b]<br>6: New patient endocrinology[a]<br>7: Consultation endocrinology[a]<br>8: Group consultation endocrinology[a]<br>9: DEXA scan[a]<br>10: Labs[a]<br>11: Consultation fertility[a]
Date of appointments/frequency	Medical record	Scale	N/A
Duration of appointments	Medical record	Scale	Time in minutes
Patient attendance	Medical record	Nominal	1: Attended<br>2: Cancelled<br>3: Did not attend
Clinician attendance**	Medical record	Nominal	1: Psychologist<br>2: Psychologist assistant<br>3: Psychiatrist<br>4: Endocrinologist<br>5: Doctors assistant<br>6: Nurse

For peer review only

LOGIC retrospective analysis protocol

	7: Gynaecologist<br>8: Sexologist<br>9: Doctor<br>10: Unknown

*Data available for T&P NHSFT only
**Data available for AUMC only

Note. Appointment type groupings: [a]Endocrinology/Medical; [b]Assessment; [c]Psychosocial Treatment; [d]Group

For peer review only

Appendix 1. Information regarding appointment types in the specialist gender clinics

	Type of clinical appointment	Definition
T&P NHSFT		
	1: Early Liaison with endocrine clinic for <15 yrs[a]	Appointment with the endocrinology clinic for those aged 15+ years
	2: Liaison with endocrine clinic for 15+ yrs[a]	Appointment with the endocrinology clinic for those aged <15 years
	3: GIDS Outreach Assessment[b]	Assessment undertaken remotely from the main clinic base in London
	4: GIDS Standard Assessment[b]	Assessment which took place at the London clinic
	5: GIDS Treatment Outreach[c]	Psychosocial treatment undertaken remotely from the main clinic base in London
	6: GIDS Treatment Standard[c]	Psychosocial treatment which took place at the London clinic
	7: GIDS Young Persons Group[d]	Group appointment specifically for young people (take place during GIDS group sessions)
	8: Group (not specified)[d]	Group appointment (take place during GIDS group sessions)
	9: GIDS Transitions Appointment[c]	Appointment for young people with GIDS and GIC clinician to discuss transition to adult service
	10: Endocrinology (15+ yrs)[a]	Appointment with the endocrinology clinic for those aged 15+ years
	11: Endocrinology (<15 yrs)[a]	Appointment with the endocrinology clinic for those aged <15 years
	12: Child, Young Adults and Families (CYAF) Assessment[b]	Assessment by CYAF clinician
	13: CYAF Individual therapy once per week[c]	Individual therapy by a CYAF clinician
	14: CYAF Family Therapy[c]	Family therapy by a CYAF clinician
AUMC		
	1: Intake Psychology[b]	First GID appointment with the psychologist during the diagnostic phase, 60 min
	2: Consultation Psychology[b]	Standard GID assessment with the psychologist during the diagnostic phase, 60 min

For peer review only

3: Psychological Assessment[b]	Appointment for completing questionnaires & psychiatric assessment / interview during the diagnostic phase, 2-3h
4: Screening Psychiatry[b]	First appointment after referral to the AUMC is with the main practitioner / treatment provider<br>For children/adolescents the appointed main practitioner in the hospital is the Child and Adolescent psychiatrist<br>After the psychiatric screening, the psychiatrist refers the CYP to a psychologist for the GID assessment / diagnostic phase
5: Consultation Psychiatry[b]	Psychiatric assessment during the diagnostic phase<br>Usually upon request from psychologist (for example in case when CYP presents with (complex) psychiatric comorbidity)
6: New patient endocrinology[a]	First appointment with the endocrinology clinic for CYP
7: Consultation endocrinology[a]	Standard appointment with the endocrinology clinic for CYP
8: Group consultation endocrinology / Webinar[a]	Group consultation before start medical treatment (providing education/information re medical treatment)
9: DEXA scan[a]	Order for DEXA scan to check bone health
10: Labs[a]	Appointment with the nurse for bloods / weight / height measurements and standard health checks
11: Consultation fertility[a]	Appointment with the fertility doctor (GYN)

Note. Appointment type groupings: [a]Endocrinology/Medical; [b]Assessment; [c]Psychosocial Treatment; [d]Group

For peer review only

Appendix 2. Information regarding care pathways

In the UK, following the NHS England service specification, all patients attend the service for assessment in the first instance. According to the service specifications, this typically consists of 3-6 appointments, with the number of appointments based on individual need, agreed with the young person and their family. At the end of the assessment, if the young person remains in contact with the service they either continue to explore their gender identity and options around this, or they may be referred to the endocrine clinic with ongoing exploration of gender and pathways and psychosocial support. The current NHS service specification can be found here: https://www.england.nhs.uk/wp-content/uploads/2017/04/gender-development-service-children-adolescents.pdf. Please note that this is currently undergoing a scheduled review, and an amendment was made to the service specification in December 2020 following the Bell vs. Tavistock judicial review. The amendment can be found here: https://www.england.nhs.uk/wp-content/uploads/2020/12/Amendment-to-Gender-Identity-Development-Service-Specification-for-Children-and-Adolescents.pdf

As GIDS is a national service, ‘outreach’ assessment simply refers to the assessment being undertaken remotely from the main clinic base in London. After an assessment, if the CYP remains in contact with the service, appointment type then changes to ‘treatment’: ‘treatment outreach’ would indicate the treatment took place outside the main London clinic base, whereas ‘treatment standard’ would mean that the treatment took place at the London clinic. We will certainly provide additional descriptions when reporting findings.

In the Netherlands, the first consultation (‘intake’) is always with a child and adolescent psychiatrist. The following appointments during the assessment phase are with a psychologist. This usually consists of 3-6 appointments (on a monthly basis), with the number of appointments based on individual need, and agreed with the young person and

For peer review only

their family. At the end of the assessment, the majority of CYP are referred to endocrinology and also continue to see a psychologist for psychosocial support. The young person and their family are seen by the endocrinologist and psychologist every 3 months.

For peer review only
